# Number and function of peripheral blood endothelial progenitor cells in Henoch-Schönlein purpura nephritis children with different degrees of renal vascular lesions

**DOI:** 10.3892/etm.2012.863

**Published:** 2012-12-18

**Authors:** XI-QIANG DANG, XIAO-JIE HE, HAI-XIA CHEN, QING-NAN HE, ZHU-WEN YI

**Affiliations:** Laboratory of Pediatric Nephrology, The Second Xiangya Hospital, Central South University, Changsha, Hunan 410011, P.R. China

**Keywords:** Henoch-Schönlein purpura nephritis, endothelial progenitor cells, blood vessels, pathology, children

## Abstract

The aim of this study was to explore the correlation between different degrees of renal vascular lesions in children with Henoch-Schönlein purpura nephritis (HSPN) and changes in progenitor cell number and function in peripheral blood. Forty-eight HSPN patients were divided into three groups, mild, moderate and severe, according to the degree of renal vascular lesions. Peripheral blood mononuclear cells were isolated and cultured. Endothelial progenitor cells (EPCs) were identified by immunofluorescence assay. The number of EPCs and the migration and adhesion of EPCs were detected by flow cytometry. The numbers of peripheral blood CD34^+^, kinase insert domain receptor^+^ (KDR^+^) and CD133^+^ cells were lower in the severe and moderate vascular lesion groups compared with the mild vascular lesion group (all P<0.05) and were also lower in the severe vascular lesion group compared with the mild and moderate vascular lesion groups (all P<0.05). The adhesion and migration of EPCs were reduced in turn in the mild, moderate and severe groups. There were significant differences between the severe group and the mild and moderate groups (all P<0.05). Renal vascular lesions are involved in the occurrence and development of HSPN, while the number of EPCs, migration and adhesion of EPCs are important factors in renal vascular lesions.

## Introduction

Henoch-Schönlein purpura nephritis (HSPN) is the most common secondary renal disease in children and its morbidity is only less than that of primary nephrotic syndrome and acute glomerulonephritis. Vasculitis is a clinical manifestation of HSPN and it has been shown that renal vascular lesions are significant in HSPN (1). The mechanism may be endothelial cell damage leading to reduced renal capillary density and the local occurrence of chronic ischemic changes in the kidney, thereby increasing renal pathological damage (2). The discovery of circulating endothelial progenitor cells (EPCs) by Asahara *et al*(3,4), and further data suggesting their participation in postnatal vasculogenesis, provides evidence for vascular regeneration. EPCs multiply and differentiate into vascular endothelial cells, which facilitate vascular repair (5). Studies have revealed that EPCs differentiate into mature endothelial cells, involved in the repair of damaged endothelial cells. A decrease in this process and functional damage may induce vascular lesions and dysfunctional regeneration (6). A previous study (7) revealed that levels of EPCs in the circulation are indicative of risk for vascular disease. Patients with the highest number of circulating EPCs were least at risk of coronary artery disease, suggesting that circulating EPC levels and the maintenance of vascular integrity are associated and may be of major clinical relevance (8). Recent studies demonstrated the presence of bone marrow-derived EPCs in the systemic circulation. They increase in number in response to certain cytokines and/or tissue ischemia and they target and are incorporated into the site of neovascularization (9,10). Evidence indicates that the number and function of EPCs are related to blood vessel damage and repair. In patients with chronic kidney disease (CKD), a decrease in circulating EPCs may impair vascular regenerative potential and thus contribute to a higher cardiovascular risk. The effect of significantly increased endostatin levels on the endothelial function and progenitors in patients with CKD requires further investigation (11–15). In the process of HSPN development, the correlation between EPCs and renal vascular lesions in HSPN patients has not been reported, but findings confirming that EPCs take part in vascular endothelial regeneration suggest that the progression of HSPN may result from insufficient delivery or decreased production of EPCs. In the current study, we monitored changes to EPC number, migration and adhesion function in the peripheral blood of HSPN patients with different vascular lesions and the correlation between EPCs and renal vascular lesions to provide information useful in the clinical diagnosis and treatment of HSPN.

## Materials and methods

### Research subjects

Forty-eight children with HSPN, diagnosed by clinical manifestation and renal biopsy, were observed between June 2004 and August 2009 in the Second Xiangya Hospital of Central South University, China. All subjects conformed to the standard set out by the national pediatric renal disease team (16). There were 29 males and 19 females, aged 2.2–14.2 years. Of the 48 HSPN patients, 19 patients had hematuria (39.6%), including 9 patients with gross hematuria, 5 patients with microscopic hematuria and 5 patients with isolated hematuria; 17 patients had isolated proteinuria (35.4%) with serum albumin lower than normal and 9 patients presented nephrotic syndrome (18.8%); and 5 patients had typical symptoms of Henoch-Schönlein purpura, including skin purpura, and an abnormal renal biopsy, despite the urine routine being normal. The course of the disease was 2 months to 6 years. According to the degree of vascular pathology, the 48 HSPN patients were divided into three groups: mild, moderate and severe. Twenty healthy patients were simultaneously set as the control group.

### Renal biopsy pathological examination

Tissues from the renal biopsy were examined by light microscopy and immunofluorescence. For the light microscopic examination, tissues were embedded in Petroline, sliced to 2 *μ*m thickness and stained by hematoxylin and eosin (H&E), periodic acid-Schiff (PAS), periodic acid-Schiff metheramine (PASM) and Masson’s staining. Immunoglobulin (Ig)-A, IgG, IgM, C1q, C3 and C4 were detected in the frozen sections by direct immunofluorescence. Glomerular lesions were classified using International Society for Kidney Disease Community (ISKDC) guidelines and HSPN was classified into levels I–VI. Vascular lesion degree was evaluated by the method of Katafuchi *et al*(17). Each sample included >3 renal interstitium vessels (artery) and the condition of vascular lesions, including the presence or absence of vessel wall thickening, hardening and hyalinization, was observed. As long as one vessel demonstrated these changes, it was defined as having vessel lesions. Vessel wall thickening was defined as vessel inner diameter/outer diameter <0.5 in the cross-section. The scores allocated according to the percentage of lesioned vessels were 0, 1 (<10%), 2 (10–25%) and 3 (>25%). The vessel wall thickening/hardening/hyalinizating was also scored from 0 to 3. The total score was calculated by adding both scores together; score 0, no vessel lesions; score 1–2, mild vessel lesions; score 3–4, moderate vessel lesions and score 5–6, severe vessel lesions. The 48 HSPN patients were divided into mild, moderate and severe groups according to the degree of vessel pathology.

### EPC isolation, culture and identification

We extracted 5 ml peripheral blood, isolated the mononuclear cells and inoculated them into a 20-hole culture board coated with human fibronectin (HFN). The cells were cultivated in endotheliocyte basic medium (EBM)-2 with 20% fetal bovine serum (FBS), 50 *μ*g/l vascular endothelial growth factor (VEGF), 50 *μ*g/l stem cell growth factor (SCF), 100 U/ml penicillin and 100 U/ml phytomycin and were placed in a 37°C CO_2_ incubator. After 4 days, we exchanged the medium and removed the non-adherent cells. After 7 days, the adherent cells were mixed with DiI-low density lipoprotein (LDL), with a final concentration of 24 mg/l. They were incubated for 1 h at 37°C, then fixed with 4% paraformaldehyde. Fluorescein isothiocyanate-labeled *Ulex europaeus* agglutin-I (FITC-UEA-I) was added (final concentration, 10 mg/l) and placed in an incubator for 1 h at 37°C. Finally we viewed and counted the cells under an inverted microscope. Cells stained positive with DiI-LDL and FITC-UEA-I were differentiating vascular EPCs (18). At the same time, adherent cells were detected for phycoerythrin (PE)-CD34, PE-CD133 and PE-kinase insert domain receptor (PE-KDR) expression by flow cytometry, controlled by the corresponding PE-IgG1.

### EPC adhesion and migration

We used trypsin to digest the adherent cells, which were then collected, added to EBM-2 medium (including 5% FBS), counted, then inoculated onto a culture board coated with HFN. The cells were left to culture for 30 min in a 37°C incubator. We then washed out non-adherent cells with phosphate-buffered saline (PBS) and counted the number of adherent cells (magnification, ×200) (19). For the detection of EPC migration, we collected adherent cells, added them to EBM-2 medium and counted them. EBM-2 medium and VEGF (50 *μ*g/ml) were added to the inferior chamber of the modified Boyden chamber and 2×10^4^ EPCs suspended in 50 *μ*l medium were added to the superior chamber. After cultivating for 24 h, non-moving cells on the filter membrane were removed. All other cells were fixed by methanol, stained by Giemsa, then three fields were selected randomly and cells that had migrated to the underlayer were counted (magnification, ×200) (20).

### Statistical analysis

We used SPSS (SPSS Inc., Chicago, IL, USA) for Windows 10.0 to analyze data. Numeration data are presented as mean ± standard deviation. Data were compared between the groups and between several points in the same group. Homogeneity of variance was analyzed using a one-way analysis of variance (ANOVA), heterogeneity of variance was analyzed using Kruskal-Wallis and least significant difference (LSD) and Mann-Whitney U tests were used to compare data between two groups. P<0.05 was considered to indicate a statistically significant difference.

## Results

### Pathological types and vascular lesion levels of HSPN patients

**Figure 1.**
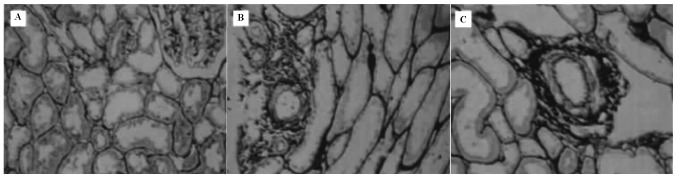
Different vascular lesions (magnification, ×200, periodic acid-silver metheramine). (A) The mild vascular lesion group; (B) the moderate vascular lesion group; (C) the severe vascular lesion group.

According to glomerular pathological levels, 11 patients were level II, 21 patients were level III, 12 patients were level IV and 4 patients were level V. All glomerular mesenteria in the HSPN patients had deposits of IgA of different degrees. According to the degree of vascular lesions, 21 patients belonged to the mild group (1.512±0.306; semi-quantitative score of vascular damage); 20 patients belonged to the moderate group (3.517±0.468) and 7 patients belonged to the severe group (5.416±0.367). Compared with the mild group, pathological integrations in the moderate and severe groups significantly increased (both P<0.01) and pathological integration in the severe group was significantly higher than in the moderate group (P<0.01). We demonstrated that the more severe the renal lesion, the more severe the vascular lesion (Table I and Fig. 1).

### Number of peripheral blood EPCs in HSPN patients with different degrees of renal vascular lesions

After being cultured *in vitro* for 7 days, EPCs in peripheral blood changed into endothelioid cells with a spindle-shape. Under a fluorescent inverted microscope, cells phagocytizing Dil-LDL evoked a red fluorescence and cells integrating FITC-UEA-I evoked a green fluorescence. Cells revealing a positive staining for both expressed a yellow fluorescence, which confirmed that those adherent cells were differentiating EPCs. The numbers of CD34^+^, KDR^+^ and CD133^+^ cells were lower in the moderate and severe vascular lesion groups than that in the control group (all P<0.05). The numbers of CD34^+^ and CD133^+^ cells were higher in the mild vascular lesion group than in the control group and of KDR^+^ cells were lower in the mild group than in the control group; however, these differences had no statistical significance (all P>0.05). With the exception of KDR in the moderate vascular lesion group, the numbers of CD34^+^, KDR^+^ and CD133^+^ cells were significantly lower in the moderate and severe vascular lesions groups than in the mild vascular lesion group (all P<0.05). The numbers of CD34^+^, KDR^+^ and CD133^+^ cells were lower in the severe vascular lesion group than in the mild and moderate vascular lesions groups (all P<0.05; Table II).

### Adhesion and migration of peripheral blood EPCs in HSPN patients with different degrees of renal vascular lesions

The adhesion and migration activity of EPCs was higher in the control group than in the mild, moderate and severe groups; however, the difference between the mild and control groups had no statistical significance (Table III). The adhesion and migration activities of EPCs were reduced in turn in the mild, moderate and severe groups. There were significant differences between the severe group and the mild and moderate groups (all P<0.05). The adhesion and migration activities of EPCs in the moderate group were lower than in the mild group; however, the difference had no statistical significance.

## Discussion

Vasculitis is a clinical manifestation of HSPN. The pathogenesis may be that the impaired vascular endothelial cells induce a reduction in the renal micrangium density, causing the kidneys to develop chronic ischemia and renal pathological lesions to worsen (2). In this study, we observed that the more severe the renal pathological lesions are, the worse the vascular lesions are, which demonstrates that renal vascular lesions play an important role in the occurrence and development of HSPN. This may be due to the vascular lesions of the renal interstitium inducing renal interstitial ischemia and hypoxia, resulting in an infiltration of inflammatory cells. Inflammatory cytokines and mediators are released and fibrocytes proliferate, promoting renal tubular epithelial cell apoptosis, renal tubular atrophy, and an increase in the rate of development of renal interstitial fibrosis (21,22). Instantaneously, renal interstitial vascular lesions increase the resistance of glomerular blood vessels, affecting blood supply to the glomeruli. This induces further damage to the glomeruli and renal interstitium, resulting in a cyclic process of damage between vessels, glomeruli and the renal tubular interstitium. Previous studies (23) have considered that immune damage, immune-medium and metabolic abnormalities are the initiating agents of HSPN vascular lesions. Further research has shown that vascular epithelial cell damage and the resultant cell number decrease, induced by various agents, are the most direct influential factors (24).

EPCs are a group of precursor cells that multiply and differentiate into mature vascular endothelial cells and are involved in postnatal vascular growth and the repair of endothelial damage. Twenty-five percent of epithelial cells in the newborn vessels are differentiated from EPCs (25). Recent research demonstrates that there are a number of EPCs in the peripheral blood, and when cytokine irritation and local ischemia occur, EPCs mobilize to the impaired site to aid in vascular regrowth (26) and the repair of the vascular endothelium. Vascular endothelial repair is a complicated regulated process, including mobilization, adhesion, chemo-taxis, migration, invasion to the ischemic tissue gaps and then differentiation to mature vascular endothelium cells and the formation of new vessels (27). In the current study, we found that the more severe the renal vascular lesion, the fewer the number of EPCs in the peripheral blood, suggesting that there is a correlation between the decrease in EPC number and renal vascular lesions in HSPN patients. However, in the mild group, the numbers of CD34^+^ and CD133^+^ cells were higher than in the control group, which may be a reverse feedback mechanism in the stress repair of autogenous vascular lesions. KDR is a type of event marker for when EPCs differentiate into endothelial cells. The KDR count in the peripheral blood was less in the mild group of HSPN patients than in the normal group. This indicates that EPCs recruited by stress recovery are not able to supply enough endothelial cells in the case of vascular damage. We found that EPC adhesion and migration activity in the peripheral blood of the control group was higher than in the mild, moderate and severe groups and EPC adherence and migration were in turn decreased in the mild, moderate and severe groups. This suggests that the renal vascular regrowth and endothelial repair are related not only to the number of EPCs, but also to the decrease of adhesion and migration activities.

In this study we found that renal vascular lesions are involved in the occurrence and development of HSPN and the number of EPCs, as well as the migration and adhesion of EPCs, are important factors in renal vascular lesions.

## Figures and Tables

**Table I.** Renal pathological levels (II–V) of groups with different degrees of vascular lesions (n).

Groups	Level II	Level III	Level IV	Level V	Total
Mild group	9	12	0	0	21
Moderate group	2	8	9	1	20
Severe group	0	1	3	3	7
All	11	21	12	4	48

**Table II.** Comparison of EPC counts in the peripheral blood of HSPN patients with different degrees of vascular lesions.

Groups	Number	CD34^+^ count	CD133^+^ count	KDR^+^ count
Mild group	21	57.08±8.25	31.14±5.66	46.14±8.23
Moderate group	20	45.16±4.38^a,c^	21.47±2.79^a,c^	39.47±7.82^a^
Severe group	7	38.96±3.74^b,^^d^	11.79±2.01^b,^^d,^^e^	27.56±5.64^b,^^f^
Control group	20	53.37±6.41	30.21±5.36	48.35±9.03
F-value		85.79	121.47	46.38
P-value		0.00	0.00	0.00

a P<0.05,

b P<0.01, compared with the control group;

c P<0.05,

d P<0.01, compared with the mild vascular lesion group;

e P<0.05,

f P<0.01, compared with the moderate vascular lesion group. Data is represented as mean ± standard deviation. EPC, endothelial progenitor cell; HSPN, Henoch-Schönlein purpura nephritis; KDR, kinase insert domain receptor.

**Table III.** Comparison of adhesion and migration of peripheral blood EPCs in HSPN children with different degrees of renal vascular lesions.

Groups	Number	Adhesion function	Migration function
Mild group	21	23.25±2.35	12.17±2.38
Moderate group	20	18.61±3.09^a^	10.19±2.79^a^
Severe group	7	12.47±2.63^b,^^c,^^e^	8.69±2.20^a,d,e^
Control group	20	25.47±2.79	14.56±2.25
F-value		76.47	63.65
P-value		0.00	0.00

a P<0.05,

b P<0.01, compared with the normal group;

c P<0.01,

d P<0.05 compared with the mild vascular lesion group;

e P<0.05, compared with the moderate vascular lesion group. Data is represented as mean ± standard deviation. EPCs, endothelial progenitor cells; HSPN, Henoch-Schönlein purpura nephritis.
